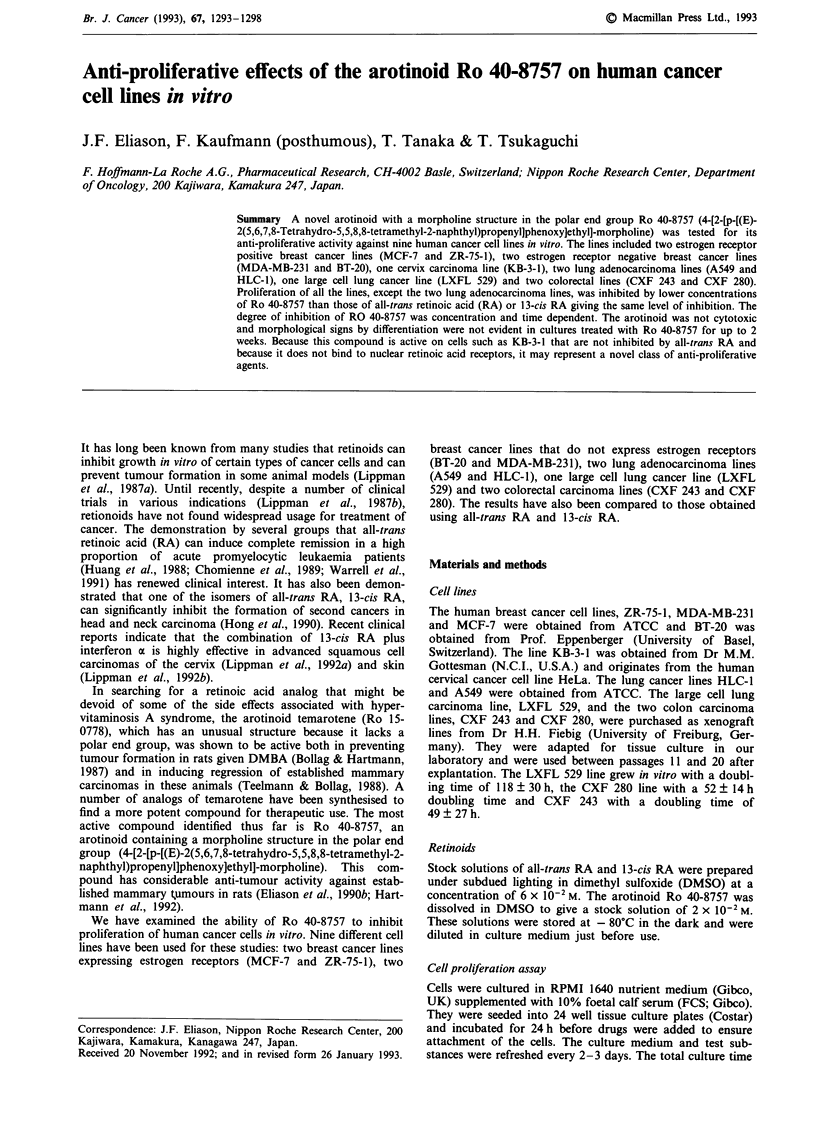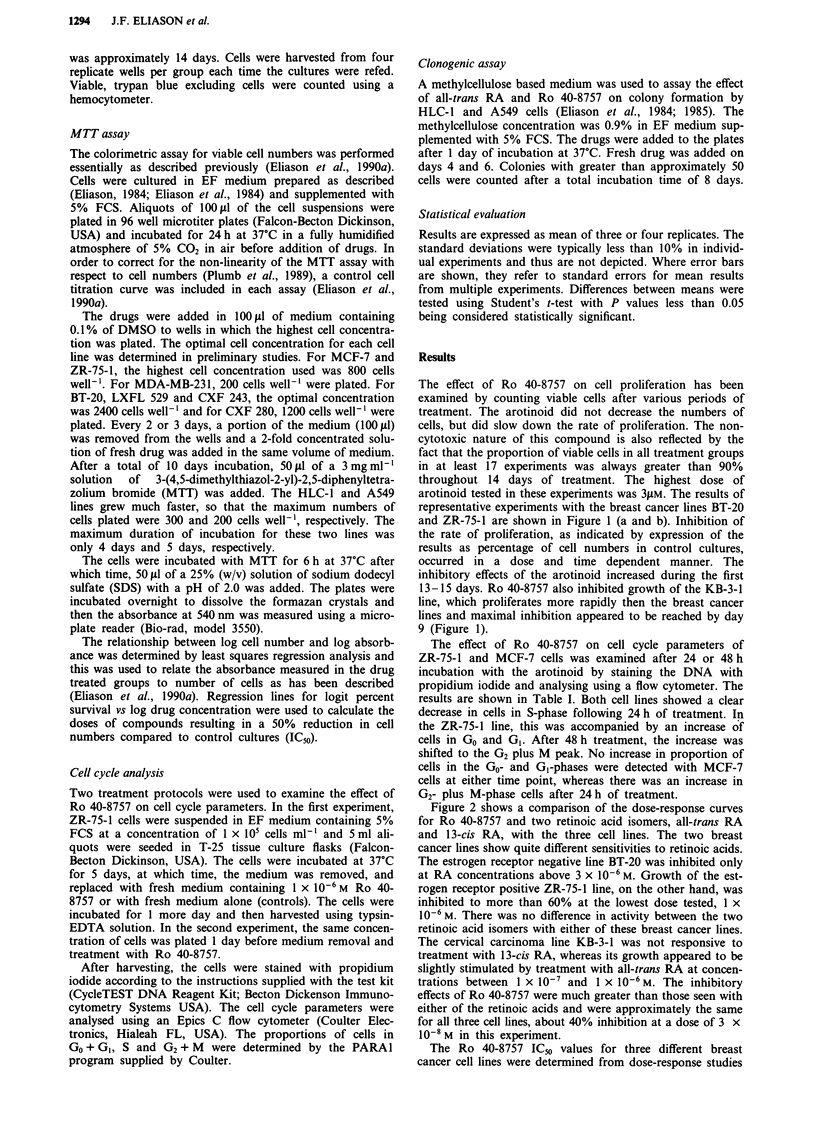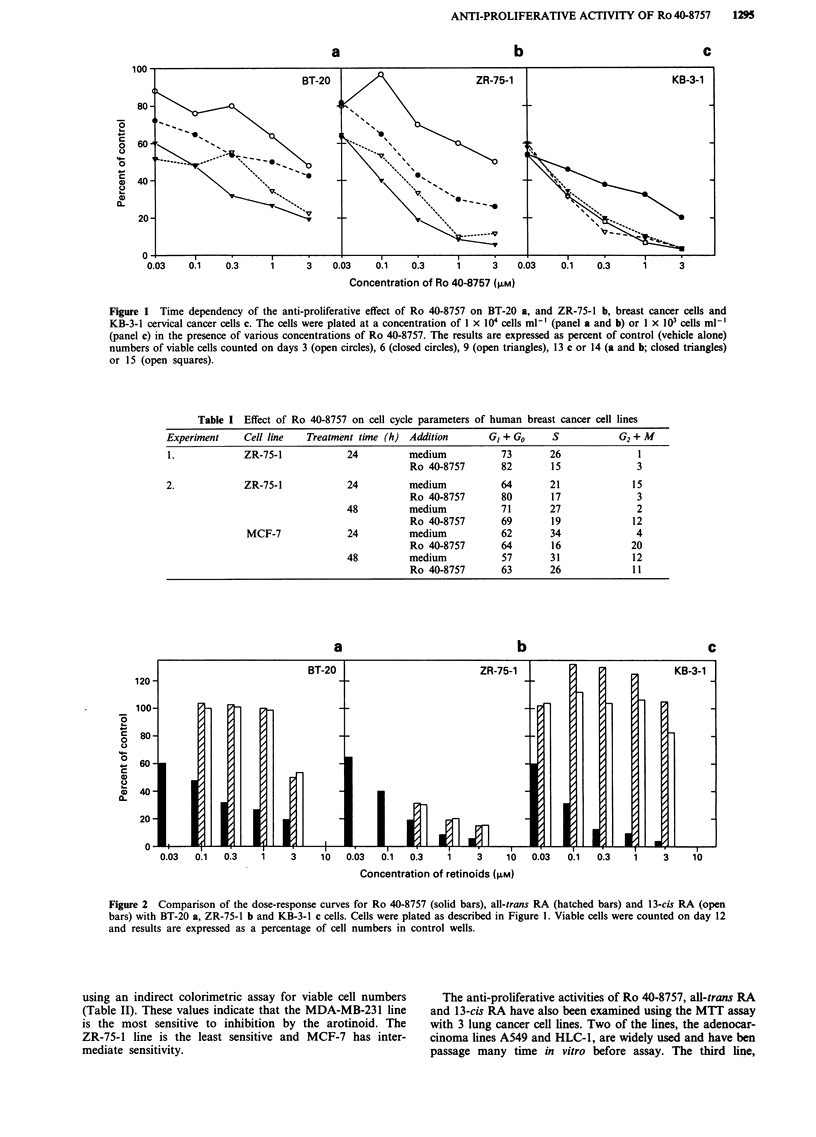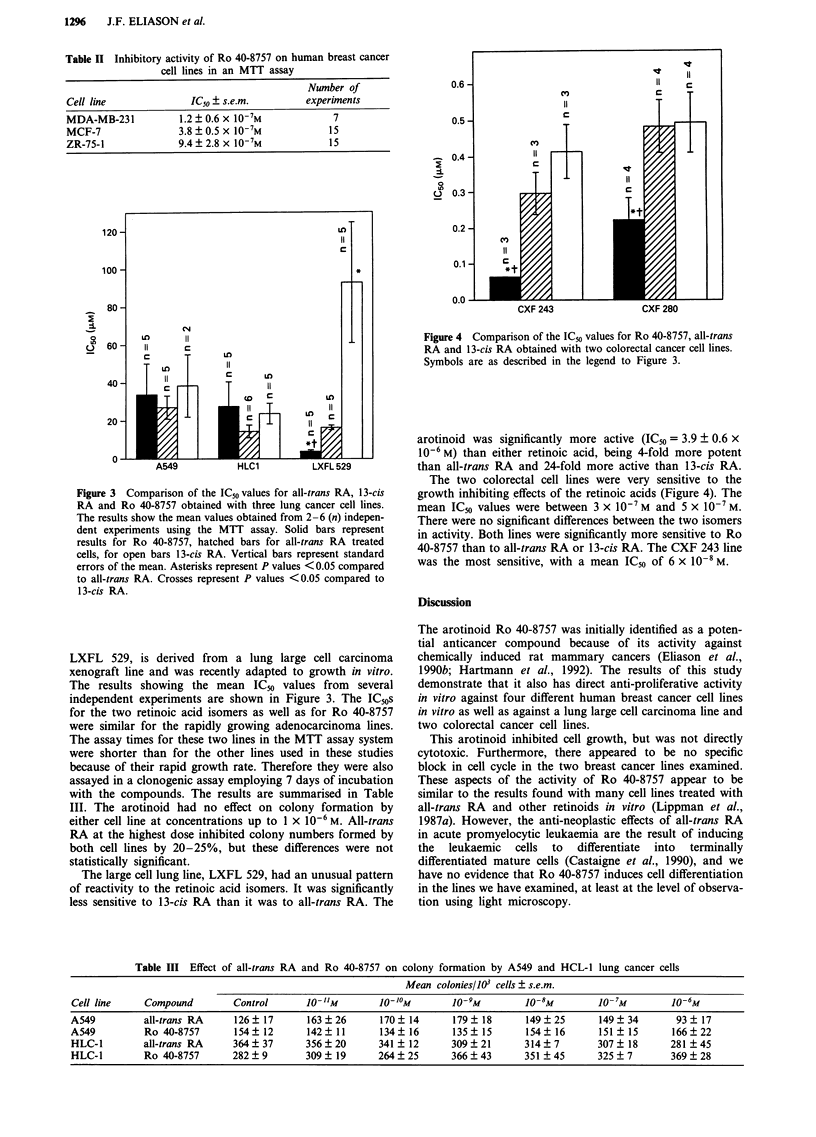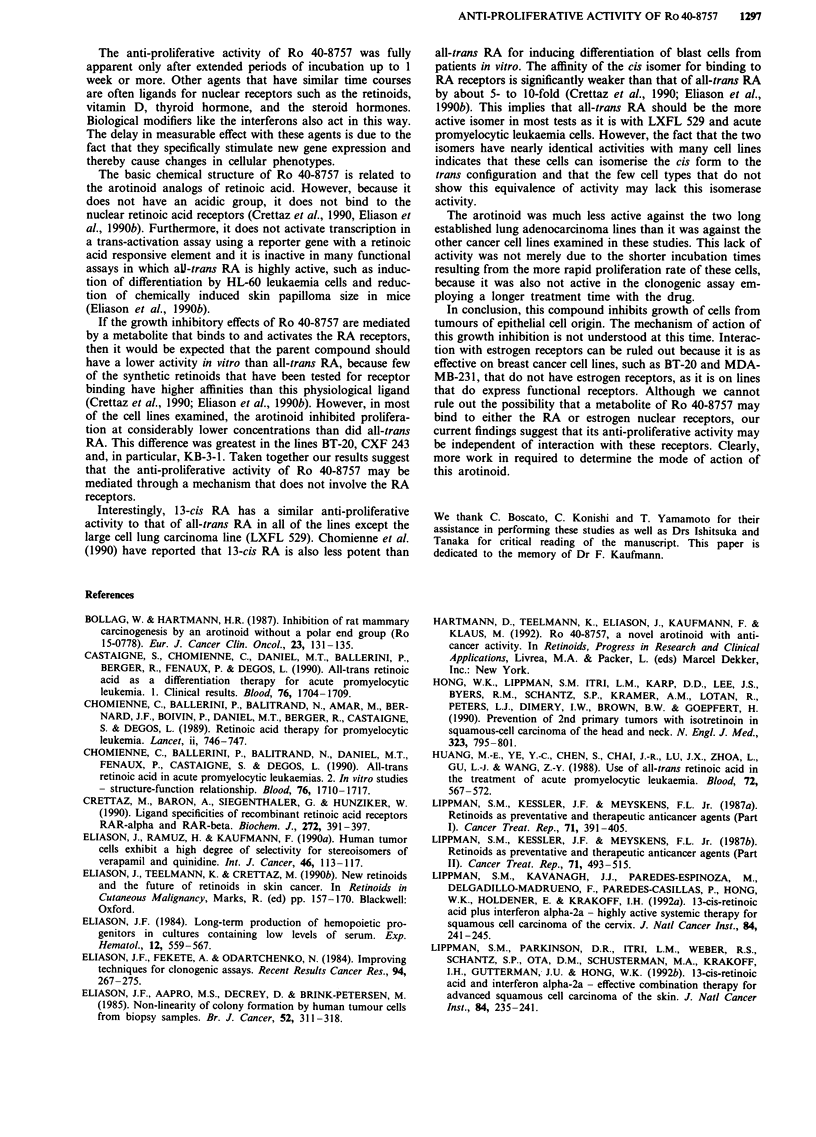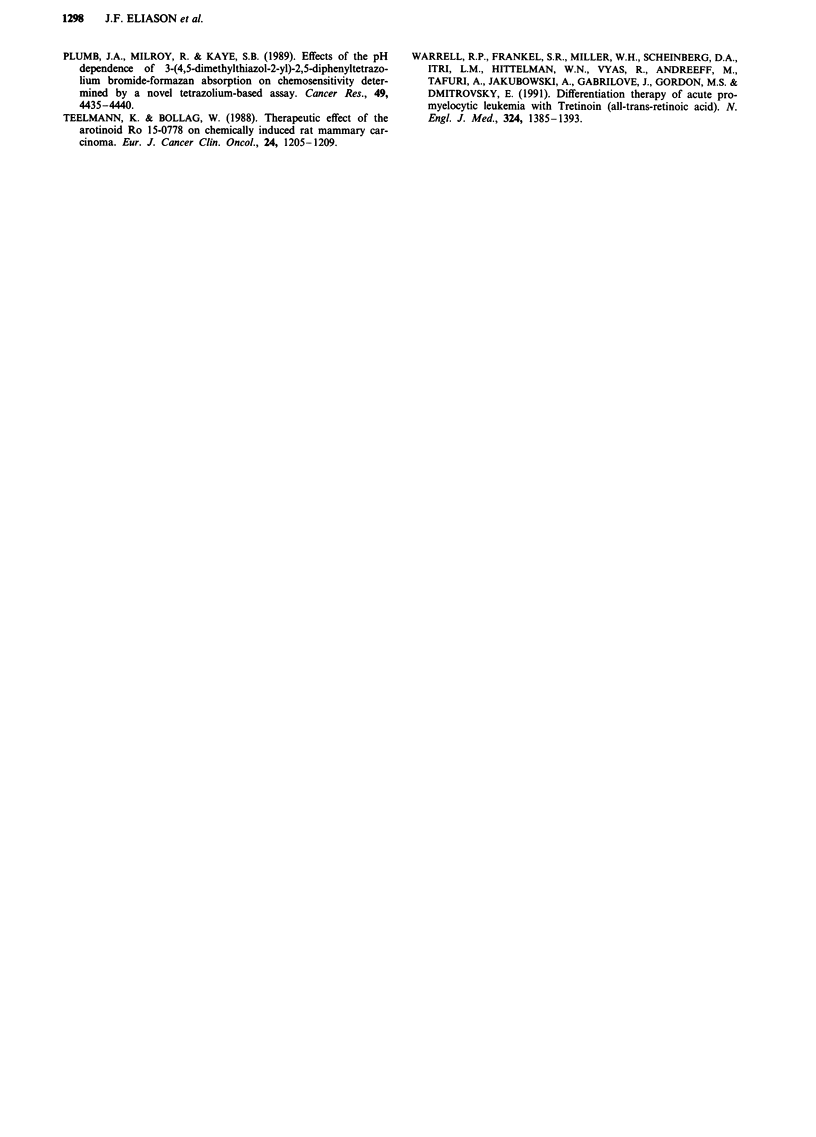# Anti-proliferative effects of the arotinoid Ro 40-8757 on human cancer cell lines in vitro.

**DOI:** 10.1038/bjc.1993.240

**Published:** 1993-06

**Authors:** J. F. Eliason, F. Kaufmann, T. Tanaka, T. Tsukaguchi

**Affiliations:** F. Hoffmann-La Roche A.G., Pharmaceutical Research, Basle, Switzerland.

## Abstract

A novel arotinoid with a morpholine structure in the polar end group Ro 40-8757 (4-[2-[p-[(E)-2(5,6,7,8-Tetrahydro-5,5,8,8-tetramethyl-2- naphthyl)propenyl]phenoxy]ethyl]-morpholine) was tested for its anti-proliferative activity against nine human cancer cell lines in vitro. The lines included two estrogen receptor positive breast cancer lines (MCF-7 and ZR-75-1), two estrogen receptor negative breast cancer lines (MDA-MB-231 and BT-20), one cervix carcinoma line (KB-3-1), two lung adenocarcinoma lines (A549 and HLC-1), one large cell lung cancer line (LXFL 529) and two colorectal lines (CXF 243 and CXF 280). Proliferation of all the lines, except the two lung adenocarcinoma lines, was inhibited by lower concentrations of Ro 40-8757 than those of all-trans retinoic acid (RA) or 13-cis RA giving the same level of inhibition. The degree of inhibition of RO 40-8757 was concentration and time dependent. The arotinoid was not cytotoxic and morphological signs by differentiation were not evident in cultures treated with Ro 40-8757 for up to 2 weeks. Because this compound is active on cells such as KB-3-1 that are not inhibited by all-trans RA and because it does not bind to nuclear retinoic acid receptors, it may represent a novel class of anti-proliferative agents.


Br. J. Cancer (1993), 67, 1293–1298 © Macmillan Press Ltd., 1993

### Anti-proliferative effects of the arotinoid Ro 40-8757 on human cancer cell lines *in vitro*

J.F. Eliason, F. Kaufmann (posthumous), T. Tanaka & T. Tsukaguchi

*F. Hoffmann-La Roche A.G., Pharmaceutical Research, CH-4002 Basle, Switzerland; Nippon Roche Research Center, Department of Oncology, 200 Kajiwara, Kamakura 247, Japan.*

**Summary** A novel arotinoid with a morpholine structure in the polar end group Ro 40-8757 (4-[2-[p-[(E)-2(5,6,7,8-Tetrahydro-5,5,8,8-tetramethyl-2-naphthyl)propenyl]phenoxy]ethyl]-morpholine) was tested for its anti-proliferative activity against nine human cancer cell lines *in vitro*. The lines included two estrogen receptor positive breast cancer lines (MCF-7 and ZR-75-1), two estrogen receptor negative breast cancer lines (MDA-MB-231 and BT-20), one cervix carcinoma line (KB-3-1), two lung adenocarcinoma lines (A549 and HLC-1), one large cell lung cancer line (LXFL 529) and two colorectal lines (CXF 243 and CXF 280). Proliferation of all the lines, except the two lung adenocarcinoma lines, was inhibited by lower concentrations of Ro 40-8757 than those of all-*trans* retinoic acid (RA) or 13-*cis* RA giving the same level of inhibition. The degree of inhibition of RO 40-8757 was concentration and time dependent. The arotinoid was not cytotoxic and morphological signs by differentiation were not evident in cultures treated with Ro 40-8757 for up to 2 weeks. Because this compound is active on cells such as KB-3-1 that are not inhibited by all-*trans* RA and because it does not bind to nuclear retinoic acid receptors, it may represent a novel class of anti-proliferative agents.

It has long been known from many studies that retinoids can inhibit growth *in vitro* of certain types of cancer cells and can prevent tumour formation in some animal models (Lippman *et al.*, 1987*a*). Until recently, despite a number of clinical trials in various indications (Lippman *et al.*, 1987*b*), retionoids have not found widespread usage for treatment of cancer. The demonstration by several groups that all-*trans* retinoic acid (RA) can induce complete remission in a high proportion of acute promyelocytic leukaemia patients (Huang *et al.*, 1988; Chomienne *et al.*, 1989; Warrell *et al.*, 1991) has renewed clinical interest. It has also been demonstrated that one of the isomers of all-*trans* RA, 13-*cis* RA, can significantly inhibit the formation of second cancers in head and neck carcinoma (Hong *et al.*, 1990). Recent clinical reports indicate that the combination of 13-*cis* RA plus interferon α is highly effective in advanced squamous cell carcinomas of the cervix (Lippman *et al.*, 1992*a*) and skin (Lippman *et al.*, 1992*b*).

In searching for a retinoic acid analog that might be devoid of some of the side effects associated with hypervitaminosis A syndrome, the arotinoid temarotene (Ro 15-0778), which has an unusual structure because it lacks a polar end group, was shown to be active both in preventing tumour formation in rats given DMBA (Bollag & Hartmann, 1987) and in inducing regression of established mammary carcinomas in these animals (Teelmann & Bollag, 1988). A number of analogs of temarotene have been synthesised to find a more potent compound for therapeutic use. The most active compound identified thus far is Ro 40-8757, an arotinoid containing a morpholine structure in the polar end group (4-[2-[p-[(E)-2(5,6,7,8-tetrahydro-5,5,8,8-tetramethyl-2-naphthyl)propenyl]phenoxy]ethyl]-morpholine). This compound has considerable anti-tumour activity against established mammary tumours in rats (Eliason *et al.*, 1990*b*; Hartmann *et al.*, 1992).

We have examined the ability of Ro 40-8757 to inhibit proliferation of human cancer cells *in vitro*. Nine different cell lines have been used for these studies: two breast cancer lines expressing estrogen receptors (MCF-7 and ZR-75-1), two breast cancer lines that do not express estrogen receptors (BT-20 and MDA-MB-231), two lung adenocarcinoma lines (A549 and HLC-1), one large cell lung cancer line (LXFL 529) and two colorectal carcinoma lines (CXF 243 and CXF 280). The results have also been compared to those obtained using all-*trans* RA and 13-*cis* RA.

### Materials and methods

### *Cell lines*

The human breast cancer cell lines, ZR-75-1, MDA-MB-231 and MCF-7 were obtained from ATCC and BT-20 was obtained from Prof. Eppenberger (University of Basel, Switzerland). The line KB-3-1 was obtained from Dr M.M. Gottesman (N.C.I., U.S.A.) and originates from the human cervical cancer cell line HeLa. The lung cancer lines HLC-1 and A549 were obtained from ATCC. The large cell lung carcinoma line, LXFL 529, and the two colon carcinoma lines, CXF 243 and CXF 280, were purchased as xenograft lines from Dr H.H. Fiebig (University of Freiburg, Germany). They were adapted for tissue culture in our laboratory and were used between passages 11 and 20 after explantation. The LXFL 529 line grew *in vitro* with a doubling time of 118 ± 30 h, the CXF 280 line with a 52 ± 14 h doubling time and CXF 243 with a doubling time of 49 ± 27 h.

### *Retinoids*

Stock solutions of all-*trans* RA and 13-*cis* RA were prepared under subdued lighting in dimethyl sulfoxide (DMSO) at a concentration of $6 \times 10^{-2}$ M. The arotinoid Ro 40-8757 was dissolved in DMSO to give a stock solution of $2 \times 10^{-2}$ M. These solutions were stored at − 80°C in the dark and were diluted in culture medium just before use.

### *Cell proliferation assay*

Cells were cultured in RPMI 1640 nutrient medium (Gibco, UK) supplemented with 10% foetal calf serum (FCS; Gibco). They were seeded into 24 well tissue culture plates (Costar) and incubated for 24 h before drugs were added to ensure attachment of the cells. The culture medium and test substances were refreshed every 2–3 days. The total culture time

Correspondence: J.F. Eliason, Nippon Roche Research Center, 200 Kajiwara, Kamakura, Kanagawa 247, Japan.
Received 20 November 1992; and in revised form 26 January 1993.

was approximately 14 days. Cells were harvested from four replicate wells per group each time the cultures were refed. Viable, trypan blue excluding cells were counted using a hemocytometer.

*MTT assay*

The colorimetric assay for viable cell numbers was performed essentially as described previously (Eliason *et al.*, 1990*a*). Cells were cultured in EF medium prepared as described (Eliason, 1984; Eliason *et al.*, 1984) and supplemented with 5% FCS. Aliquots of 100 µl of the cell suspensions were plated in 96 well microtiter plates (Falcon-Becton Dickinson, USA) and incubated for 24 h at 37°C in a fully humidified atmosphere of 5% $CO_2$ in air before addition of drugs. In order to correct for the non-linearity of the MTT assay with respect to cell numbers (Plumb *et al.*, 1989), a control cell titration curve was included in each assay (Eliason *et al.*, 1990*a*).

The drugs were added in 100 µl of medium containing 0.1% of DMSO to wells in which the highest cell concentration was plated. The optimal cell concentration for each cell line was determined in preliminary studies. For MCF-7 and ZR-75-1, the highest cell concentration used was 800 cells $well^{-1}$. For MDA-MB-231, 200 cells $well^{-1}$ were plated. For BT-20, LXFL 529 and CXF 243, the optimal concentration was 2400 cells $well^{-1}$ and for CXF 280, 1200 cells $well^{-1}$ were plated. Every 2 or 3 days, a portion of the medium (100 µl) was removed from the wells and a 2-fold concentrated solution of fresh drug was added in the same volume of medium. After a total of 10 days incubation, 50 µl of a 3 mg $ml^{-1}$ solution of 3-(4,5-dimethylthiazol-2-yl)-2,5-diphenyltetrazolium bromide (MTT) was added. The HLC-1 and A549 lines grew much faster, so that the maximum numbers of cells plated were 300 and 200 cells $well^{-1}$, respectively. The maximum duration of incubation for these two lines was only 4 days and 5 days, respectively.

The cells were incubated with MTT for 6 h at 37°C after which time, 50 µl of a 25% (w/v) solution of sodium dodecyl sulfate (SDS) with a pH of 2.0 was added. The plates were incubated overnight to dissolve the formazan crystals and then the absorbance at 540 nm was measured using a microplate reader (Bio-rad, model 3550).

The relationship between log cell number and log absorbance was determined by least squares regression analysis and this was used to relate the absorbance measured in the drug treated groups to number of cells as has been described (Eliason *et al.*, 1990*a*). Regression lines for logit percent survival *vs* log drug concentration were used to calculate the doses of compounds resulting in a 50% reduction in cell numbers compared to control cultures ($IC_{50}$).

*Cell cycle analysis*

Two treatment protocols were used to examine the effect of Ro 40-8757 on cell cycle parameters. In the first experiment, ZR-75-1 cells were suspended in EF medium containing 5% FCS at a concentration of $1 \times 10^5$ cells $ml^{-1}$ and 5 ml aliquots were seeded in T-25 tissue culture flasks (Falcon-Becton Dickinson, USA). The cells were incubated at 37°C for 5 days, at which time, the medium was removed, and replaced with fresh medium containing $1 \times 10^{-6}$ M Ro 40-8757 or with fresh medium alone (controls). The cells were incubated for 1 more day and then harvested using typsin-EDTA solution. In the second experiment, the same concentration of cells was plated 1 day before medium removal and treatment with Ro 40-8757.

After harvesting, the cells were stained with propidium iodide according to the instructions supplied with the test kit (CycleTEST DNA Reagent Kit; Becton Dickenson Immunocytometry Systems USA). The cell cycle parameters were analysed using an Epics C flow cytometer (Coulter Electronics, Hialeah FL, USA). The proportions of cells in $G_0 + G_1$, S and $G_2 + M$ were determined by the PARA1 program supplied by Coulter.

*Clonogenic assay*

A methylcellulose based medium was used to assay the effect of all-*trans* RA and Ro 40-8757 on colony formation by HLC-1 and A549 cells (Eliason *et al.*, 1984; 1985). The methylcellulose concentration was 0.9% in EF medium supplemented with 5% FCS. The drugs were added to the plates after 1 day of incubation at 37°C. Fresh drug was added on days 4 and 6. Colonies with greater than approximately 50 cells were counted after a total incubation time of 8 days.

*Statistical evaluation*

Results are expressed as mean of three or four replicates. The standard deviations were typically less than 10% in individual experiments and thus are not depicted. Where error bars are shown, they refer to standard errors for mean results from multiple experiments. Differences between means were tested using Student's *t*-test with *P* values less than 0.05 being considered statistically significant.

### Results

The effect of Ro 40-8757 on cell proliferation has been examined by counting viable cells after various periods of treatment. The arotinoid did not decrease the numbers of cells, but did slow down the rate of proliferation. The non-cytotoxic nature of this compound is also reflected by the fact that the proportion of viable cells in all treatment groups in at least 17 experiments was always greater than 90% throughout 14 days of treatment. The highest dose of arotinoid tested in these experiments was 3µM. The results of representative experiments with the breast cancer lines BT-20 and ZR-75-1 are shown in Figure 1 (a and b). Inhibition of the rate of proliferation, as indicated by expression of the results as percentage of cell numbers in control cultures, occurred in a dose and time dependent manner. The inhibitory effects of the arotinoid increased during the first 13–15 days. Ro 40-8757 also inhibited growth of the KB-3-1 line, which proliferates more rapidly then the breast cancer lines and maximal inhibition appeared to be reached by day 9 (Figure 1).

The effect of Ro 40-8757 on cell cycle parameters of ZR-75-1 and MCF-7 cells was examined after 24 or 48 h incubation with the arotinoid by staining the DNA with propidium iodide and analysing using a flow cytometer. The results are shown in Table I. Both cell lines showed a clear decrease in cells in S-phase following 24 h of treatment. In the ZR-75-1 line, this was accompanied by an increase of cells in $G_0$ and $G_1$. After 48 h treatment, the increase was shifted to the $G_2$ plus M peak. No increase in proportion of cells in the $G_0$- and $G_1$-phases were detected with MCF-7 cells at either time point, whereas there was an increase in $G_2$- plus M-phase cells after 24 h of treatment.

Figure 2 shows a comparison of the dose-response curves for Ro 40-8757 and two retinoic acid isomers, all-*trans* RA and 13-*cis* RA, with the three cell lines. The two breast cancer lines show quite different sensitivities to retinoic acids. The estrogen receptor negative line BT-20 was inhibited only at RA concentrations above $3 \times 10^{-6}$ M. Growth of the estrogen receptor positive ZR-75-1 line, on the other hand, was inhibited to more than 60% at the lowest dose tested, $1 \times 10^{-6}$ M. There was no difference in activity between the two retinoic acid isomers with either of these breast cancer lines. The cervical carcinoma line KB-3-1 was not responsive to treatment with 13-*cis* RA, whereas its growth appeared to be slightly stimulated by treatment with all-*trans* RA at concentrations between $1 \times 10^{-7}$ and $1 \times 10^{-6}$ M. The inhibitory effects of Ro 40-8757 were much greater than those seen with either of the retinoic acids and were approximately the same for all three cell lines, about 40% inhibition at a dose of $3 \times 10^{-8}$ M in this experiment.

The Ro 40-8757 $IC_{50}$ values for three different breast cancer cell lines were determined from dose-response studies

**Figure 1** Time dependency of the anti-proliferative effect of Ro 40-8757 on BT-20 **a**, and ZR-75-1 **b**, breast cancer cells and KB-3-1 cervical cancer cells **c**. The cells were plated at a concentration of $1 \times 10^4$ cells $ml^{-1}$ (panel **a** and **b**) or $1 \times 10^3$ cells $ml^{-1}$ (panel **c**) in the presence of various concentrations of Ro 40-8757. The results are expressed as percent of control (vehicle alone) numbers of viable cells counted on days 3 (open circles), 6 (closed circles), 9 (open triangles), 13 **c** or 14 (**a** and **b**; closed triangles) or 15 (open squares).

**Table I** Effect of Ro 40-8757 on cell cycle parameters of human breast cancer cell lines

| *Experiment* | *Cell line* | *Treatment time (h)* | *Addition* | $G_1 + G_0$ | *S* | $G_2 + M$ |
|---|---|---|---|---|---|---|
| 1. | ZR-75-1 | 24 | medium | 73 | 26 | 1 |
| | | | Ro 40-8757 | 82 | 15 | 3 |
| 2. | ZR-75-1 | 24 | medium | 64 | 21 | 15 |
| | | | Ro 40-8757 | 80 | 17 | 3 |
| | | 48 | medium | 71 | 27 | 2 |
| | | | Ro 40-8757 | 69 | 19 | 12 |
| | MCF-7 | 24 | medium | 62 | 34 | 4 |
| | | | Ro 40-8757 | 64 | 16 | 20 |
| | | 48 | medium | 57 | 31 | 12 |
| | | | Ro 40-8757 | 63 | 26 | 11 |

**Figure 2** Comparison of the dose-response curves for Ro 40-8757 (solid bars), all-*trans* RA (hatched bars) and 13-*cis* RA (open bars) with BT-20 **a**, ZR-75-1 **b** and KB-3-1 **c** cells. Cells were plated as described in Figure 1. Viable cells were counted on day 12 and results are expressed as a percentage of cell numbers in control wells.

using an indirect colorimetric assay for viable cell numbers (Table II). These values indicate that the MDA-MB-231 line is the most sensitive to inhibition by the arotinoid. The ZR-75-1 line is the least sensitive and MCF-7 has intermediate sensitivity.

The anti-proliferative activities of Ro 40-8757, all-*trans* RA and 13-*cis* RA have also been examined using the MTT assay with 3 lung cancer cell lines. Two of the lines, the adenocarcinoma lines A549 and HLC-1, are widely used and have ben passage many time *in vitro* before assay. The third line,

**Table II** Inhibitory activity of Ro 40-8757 on human breast cancer cell lines in an MTT assay

| *Cell line* | $IC_{50}$ ± *s.e.m.* | *Number of experiments* |
|---|---|---|
| MDA-MB-231 | $1.2 \pm 0.6 \times 10^{-7}$M | 7 |
| MCF-7 | $3.8 \pm 0.5 \times 10^{-7}$M | 15 |
| ZR-75-1 | $9.4 \pm 2.8 \times 10^{-7}$M | 15 |

**Figure 3** Comparison of the $IC_{50}$ values for all-*trans* RA, 13-*cis* RA and Ro 40-8757 obtained with three lung cancer cell lines. The results show the mean values obtained from 2–6 (*n*) independent experiments using the MTT assay. Solid bars represent results for Ro 40-8757, hatched bars for all-*trans* RA treated cells, for open bars 13-*cis* RA. Vertical bars represent standard errors of the mean. Asterisks represent *P* values <0.05 compared to all-*trans* RA. Crosses represent *P* values <0.05 compared to 13-*cis* RA.

LXFL 529, is derived from a lung large cell carcinoma xenograft line and was recently adapted to growth *in vitro*. The results showing the mean $IC_{50}$ values from several independent experiments are shown in Figure 3. The $IC_{50}$s for the two retinoic acid isomers as well as for Ro 40-8757 were similar for the rapidly growing adenocarcinoma lines. The assay times for these two lines in the MTT assay system were shorter than for the other lines used in these studies because of their rapid growth rate. Therefore they were also assayed in a clonogenic assay employing 7 days of incubation with the compounds. The results are summarised in Table III. The arotinoid had no effect on colony formation by either cell line at concentrations up to $1 \times 10^{-6}$ M. All-*trans* RA at the highest dose inhibited colony numbers formed by both cell lines by 20–25%, but these differences were not statistically significant.

The large cell lung line, LXFL 529, had an unusual pattern of reactivity to the retinoic acid isomers. It was significantly less sensitive to 13-*cis* RA than it was to all-*trans* RA. The arotinoid was significantly more active ($IC_{50} = 3.9 \pm 0.6 \times 10^{-6}$ M) than either retinoic acid, being 4-fold more potent than all-*trans* RA and 24-fold more active than 13-*cis* RA.

**Figure 4** Comparison of the $IC_{50}$ values for Ro 40-8757, all-*trans* RA and 13-*cis* RA obtained with two colorectal cancer cell lines. Symbols are as described in the legend to Figure 3.

The two colorectal cell lines were very sensitive to the growth inhibiting effects of the retinoic acids (Figure 4). The mean $IC_{50}$ values were between $3 \times 10^{-7}$ M and $5 \times 10^{-7}$ M. There were no significant differences between the two isomers in activity. Both lines were significantly more sensitive to Ro 40-8757 than to all-*trans* RA or 13-*cis* RA. The CXF 243 line was the most sensitive, with a mean $IC_{50}$ of $6 \times 10^{-8}$ M.

### Discussion

The arotinoid Ro 40-8757 was initially identified as a potential anticancer compound because of its activity against chemically induced rat mammary cancers (Eliason *et al.*, 1990*b*; Hartmann *et al.*, 1992). The results of this study demonstrate that it also has direct anti-proliferative activity *in vitro* against four different human breast cancer cell lines *in vitro* as well as against a lung large cell carcinoma line and two colorectal cancer cell lines.

This arotinoid inhibited cell growth, but was not directly cytotoxic. Furthermore, there appeared to be no specific block in cell cycle in the two breast cancer lines examined. These aspects of the activity of Ro 40-8757 appear to be similar to the results found with many cell lines treated with all-*trans* RA and other retinoids *in vitro* (Lippman *et al.*, 1987*a*). However, the anti-neoplastic effects of all-*trans* RA in acute promyelocytic leukaemia are the result of inducing the leukaemic cells to differentiate into terminally differentiated mature cells (Castaigne *et al.*, 1990), and we have no evidence that Ro 40-8757 induces cell differentiation in the lines we have examined, at least at the level of observation using light microscopy.

**Table III** Effect of all-*trans* RA and Ro 40-8757 on colony formation by A549 and HCL-1 lung cancer cells

| | | *Mean colonies/$10^3$ cells ± s.e.m.* | | | | | | |
|---|---|---|---|---|---|---|---|---|
| *Cell line* | *Compound* | *Control* | $10^{-11}$M | $10^{-10}$M | $10^{-9}$M | $10^{-8}$M | $10^{-7}$M | $10^{-6}$M |
| A549 | all-*trans* RA | 126 ± 17 | 163 ± 26 | 170 ± 14 | 179 ± 18 | 149 ± 25 | 149 ± 34 | 93 ± 17 |
| A549 | Ro 40-8757 | 154 ± 12 | 142 ± 11 | 134 ± 16 | 135 ± 15 | 154 ± 16 | 151 ± 15 | 166 ± 22 |
| HLC-1 | all-*trans* RA | 364 ± 37 | 356 ± 20 | 341 ± 12 | 309 ± 21 | 314 ± 7 | 307 ± 18 | 281 ± 45 |
| HLC-1 | Ro 40-8757 | 282 ± 9 | 309 ± 19 | 264 ± 25 | 366 ± 43 | 351 ± 45 | 325 ± 7 | 369 ± 28 |

The anti-proliferative activity of Ro 40-8757 was fully apparent only after extended periods of incubation up to 1 week or more. Other agents that have similar time courses are often ligands for nuclear receptors such as the retinoids, vitamin D, thyroid hormone, and the steroid hormones. Biological modifiers like the interferons also act in this way. The delay in measurable effect with these agents is due to the fact that they specifically stimulate new gene expression and thereby cause changes in cellular phenotypes.

The basic chemical structure of Ro 40-8757 is related to the arotinoid analogs of retinoic acid. However, because it does not have an acidic group, it does not bind to the nuclear retinoic acid receptors (Crettaz *et al*., 1990, Eliason *et al*., 1990*b*). Furthermore, it does not activate transcription in a trans-activation assay using a reporter gene with a retinoic acid responsive element and it is inactive in many functional assays in which all-*trans* RA is highly active, such as induction of differentiation by HL-60 leukaemia cells and reduction of chemically induced skin papilloma size in mice (Eliason *et al*., 1990*b*).

If the growth inhibitory effects of Ro 40-8757 are mediated by a metabolite that binds to and activates the RA receptors, then it would be expected that the parent compound should have a lower activity *in vitro* than all-*trans* RA, because few of the synthetic retinoids that have been tested for receptor binding have higher affinities than this physiological ligand (Crettaz *et al*., 1990; Eliason *et al*., 1990*b*). However, in most of the cell lines examined, the arotinoid inhibited proliferation at considerably lower concentrations than did all-*trans* RA. This difference was greatest in the lines BT-20, CXF 243 and, in particular, KB-3-1. Taken together our results suggest that the anti-proliferative activity of Ro 40-8757 may be mediated through a mechanism that does not involve the RA receptors.

Interestingly, 13-*cis* RA has a similar anti-proliferative activity to that of all-*trans* RA in all of the lines except the large cell lung carcinoma line (LXFL 529). Chomienne *et al*. (1990) have reported that 13-*cis* RA is also less potent than all-*trans* RA for inducing differentiation of blast cells from patients *in vitro*. The affinity of the *cis* isomer for binding to RA receptors is significantly weaker than that of all-*trans* RA by about 5- to 10-fold (Crettaz *et al*., 1990; Eliason *et al*., 1990*b*). This implies that all-*trans* RA should be the more active isomer in most tests as it is with LXFL 529 and acute promyelocytic leukaemia cells. However, the fact that the two isomers have nearly identical activities with many cell lines indicates that these cells can isomerise the *cis* form to the *trans* configuration and that the few cell types that do not show this equivalence of activity may lack this isomerase activity.

The arotinoid was much less active against the two long established lung adenocarcinoma lines than it was against the other cancer cell lines examined in these studies. This lack of activity was not merely due to the shorter incubation times resulting from the more rapid proliferation rate of these cells, because it was also not active in the clonogenic assay employing a longer treatment time with the drug.

In conclusion, this compound inhibits growth of cells from tumours of epithelial cell origin. The mechanism of action of this growth inhibition is not understood at this time. Interaction with estrogen receptors can be ruled out because it is as effective on breast cancer cell lines, such as BT-20 and MDA-MB-231, that do not have estrogen receptors, as it is on lines that do express functional receptors. Although we cannot rule out the possibility that a metabolite of Ro 40-8757 may bind to either the RA or estrogen nuclear receptors, our current findings suggest that its anti-proliferative activity may be independent of interaction with these receptors. Clearly, more work in required to determine the mode of action of this arotinoid.

We thank C. Boscato, C. Konishi and T. Yamamoto for their assistance in performing these studies as well as Drs Ishitsuka and Tanaka for critical reading of the manuscript. This paper is dedicated to the memory of Dr F. Kaufmann.

### References

BOLLAG, W. & HARTMANN, H.R. (1987). Inhibition of rat mammary carcinogenesis by an arotinoid without a polar end group (Ro 15-0778). *Eur. J. Cancer Clin. Oncol.*, **23**, 131–135.

CASTAIGNE, S., CHOMIENNE, C., DANIEL, M.T., BALLERINI, P., BERGER, R., FENAUX, P. & DEGOS, L. (1990). All-trans retinoic acid as a differentiation therapy for acute promyelocytic leukemia. 1. Clinical results. *Blood*, **76**, 1704–1709.

CHOMIENNE, C., BALLERINI, P., BALITRAND, N., AMAR, M., BERNARD, J.F., BOIVIN, P., DANIEL, M.T., BERGER, R., CASTAIGNE, S. & DEGOS, L. (1989). Retinoic acid therapy for promyelocytic leukemia. *Lancet*, ii, 746–747.

CHOMIENNE, C., BALLERINI, P., BALITRAND, N., DANIEL, M.T., FENAUX, P., CASTAIGNE, S. & DEGOS, L. (1990). All-trans retinoic acid in acute promyelocytic leukaemias. 2. *In vitro* studies – structure-function relationship. *Blood*, **76**, 1710–1717.

CRETTAZ, M., BARON, A., SIEGENTHALER, G. & HUNZIKER, W. (1990). Ligand specificities of recombinant retinoic acid receptors RAR-alpha and RAR-beta. *Biochem. J.*, **272**, 391–397.

ELIASON, J., RAMUZ, H. & KAUFMANN, F. (1990*a*). Human tumor cells exhibit a high degree of selectivity for stereoisomers of verapamil and quinidine. *Int. J. Cancer*, **46**, 113–117.

ELIASON, J., TEELMANN, K. & CRETTAZ, M. (1990*b*). New retinoids and the future of retinoids in skin cancer. In *Retinoids in Cutaneous Malignancy*, Marks, R. (ed) pp. 157–170. Blackwell: Oxford.

ELIASON, J.F. (1984). Long-term production of hemopoietic progenitors in cultures containing low levels of serum. *Exp. Hematol.*, **12**, 559–567.

ELIASON, J.F., FEKETE, A. & ODARTCHENKO, N. (1984). Improving techniques for clonogenic assays. *Recent Results Cancer Res.*, **94**, 267–275.

ELIASON, J.F., AAPRO, M.S., DECREY, D. & BRINK-PETERSEN, M. (1985). Non-linearity of colony formation by human tumour cells from biopsy samples. *Br. J. Cancer*, **52**, 311–318.

HARTMANN, D., TEELMANN, K., ELIASON, J., KAUFMANN, F. & KLAUS, M. (1992). Ro 40-8757, a novel arotinoid with anticancer activity. In *Retinoids, Progress in Research and Clinical Applications*, Livrea, M.A. & Packer, L. (eds) Marcel Dekker, Inc.: New York.

HONG, W.K., LIPPMAN, S.M. ITRI, L.M., KARP, D.D., LEE, J.S., BYERS, R.M., SCHANTZ, S.P., KRAMER, A.M., LOTAN, R., PETERS, L.J., DIMERY, I.W., BROWN, B.W. & GOEPFERT, H. (1990). Prevention of 2nd primary tumors with isotretinoin in squamous-cell carcinoma of the head and neck. *N. Engl. J. Med.*, **323**, 795–801.

HUANG, M.-E., YE, Y.-C., CHEN, S., CHAI, J.-R., LU, J.X., ZHOA, L., GU, L.-J. & WANG, Z.-Y. (1988). Use of all-*trans* retinoic acid in the treatment of acute promyelocytic leukaemia. *Blood*, **72**, 567–572.

LIPPMAN, S.M., KESSLER, J.F. & MEYSKENS, F.L. Jr. (1987*a*). Retinoids as preventative and therapeutic anticancer agents (Part I). *Cancer Treat. Rep.*, **71**, 391–405.

LIPPMAN, S.M., KESSLER, J.F. & MEYSKENS, F.L. Jr. (1987*b*). Retinoids as preventative and therapeutic anticancer agents (Part II). *Cancer Treat. Rep.*, **71**, 493–515.

LIPPMAN, S.M., KAVANAGH, J.J., PAREDES-ESPINOZA, M., DELGADILLO-MADRUENO, F., PAREDES-CASILLAS, P., HONG, W.K., HOLDENER, E. & KRAKOFF, I.H. (1992*a*). 13-cis-retinoic acid plus interferon alpha-2a – highly active systemic therapy for squamous cell carcinoma of the cervix. *J. Natl Cancer Inst.*, **84**, 241–245.

LIPPMAN, S.M., PARKINSON, D.R., ITRI, L.M., WEBER, R.S., SCHANTZ, S.P., OTA, D.M., SCHUSTERMAN, M.A., KRAKOFF, I.H., GUTTERMAN, J.U. & HONG, W.K. (1992*b*). 13-cis-retinoic acid and interferon alpha-2a – effective combination therapy for advanced squamous cell carcinoma of the skin. *J. Natl Cancer Inst.*, **84**, 235–241.

PLUMB, J.A., MILROY, R. & KAYE, S.B. (1989). Effects of the pH dependence of 3-(4,5-dimethylthiazol-2-yl)-2,5-diphenyltetrazolium bromide-formazan absorption on chemosensitivity determined by a novel tetrazolium-based assay. *Cancer Res.*, **49**, 4435-4440.

TEELMANN, K. & BOLLAG, W. (1988). Therapeutic effect of the arotinoid Ro 15-0778 on chemically induced rat mammary carcinoma. *Eur. J. Cancer Clin. Oncol.*, **24**, 1205-1209.

WARRELL, R.P., FRANKEL, S.R., MILLER, W.H., SCHEINBERG, D.A., ITRI, L.M., HITTELMAN, W.N., VYAS, R., ANDREEFF, M., TAFURI, A., JAKUBOWSKI, A., GABRILOVE, J., GORDON, M.S. & DMITROVSKY, E. (1991). Differentiation therapy of acute promyelocytic leukemia with Tretinoin (all-trans-retinoic acid). *N. Engl. J. Med.*, **324**, 1385-1393.